# Regulatory T Cells and IL-10 Independently Counterregulate Cytotoxic T Lymphocyte Responses Induced by Transcutaneous Immunization

**DOI:** 10.1371/journal.pone.0027911

**Published:** 2011-11-16

**Authors:** Pamela Stein, Michael Weber, Steve Prüfer, Beate Schmid, Edgar Schmitt, Hans-Christian Probst, Ari Waisman, Peter Langguth, Hansjörg Schild, Markus P. Radsak

**Affiliations:** 1 Institute for Immunology, Johannes Gutenberg University Medical Center, Mainz, Germany; 2 Institute for Molecular Medicine, Johannes Gutenberg-University Medical Center, Mainz, Germany; 3 Department of Biopharmaceutics and Pharmaceutical Technology, Johannes Gutenberg University, Mainz, Germany; 4 Third Department of Medicine, Johannes Gutenberg University Medical Center, Mainz, Germany; University Paris Sud, France

## Abstract

**Background:**

The imidazoquinoline derivate imiquimod induces inflammatory responses and protection against transplanted tumors when applied to the skin in combination with a cognate peptide epitope (transcutaneous immunization, TCI). Here we investigated the role of regulatory T cells (T_reg_) and the suppressive cytokine IL-10 in restricting TCI-induced cytotoxic T lymphocyte (CTL) responses.

**Methodology/Principal Findings:**

TCI was performed with an ointment containing the TLR7 agonist imiquimod and a CTL epitope was applied to the depilated back skin of C57BL/6 mice. Using specific antibodies and FoxP3-diphteria toxin receptor transgenic (DEREG) mice, we interrogated inhibiting factors after TCI: by depleting FoxP3^+^ regulatory T cells we found that specific CTL-responses were greatly enhanced. Beyond this, in IL-10 deficient (IL-10^-/-^) mice or after blocking of IL-10 signalling with an IL-10 receptor specific antibody, the TCI induced CTL response is greatly enhanced indicating an important role for this cytokine in TCI. However, by transfer of T_reg_ in IL-10^-/-^ mice and the use of B cell deficient JHT^-/-^ mice, we can exclude T_reg_ and B cells as source of IL-10 in the setting of TCI.

**Conclusion/Significance:**

We identify T_reg_ and IL-10 as two important and independently acting suppressors of CTL-responses induced by transcutaneous immunization. Advanced vaccination strategies inhibiting T_reg_ function and IL-10 release may lead the development of effective vaccination protocols aiming at the induction of T cell responses suitable for the prophylaxis or treatment of persistent infections or tumors.

## Introduction

The development of new vaccination methods to successfully combat malignant and infectious diseases is a central aim of present studies. In this context transcutaneous immunization (TCI) approaches seem to be a promising strategy. Combining an easy-to-use administration, by avoiding the use of needles and therefore allow self-medication, and addressing the skin associated lymphoid tissue (SALT) at the same time makes TCI an attractive immunization strategy. For the induction of adaptive immune responses the administered adjuvants has to be combined with an antigen. Beside the well-studied adjuvants cholera toxin (CT) and head-labile enterotoxin (LT) [1] the imidazoquinolin imiquimod constitutes an adjuvant that passages through the epidermis without the need of any pre-treatment of the skin.

Imiquimod is a synthetic TLR7 ligand [2] that induces a wide range of inflammatory cytokines as IFN-α, TNF-α, IL-1α, IL-6 and IL-8 in immune cells like dendritic cells (DC), B cells, keratinocytes and granulocytes [3]–[7]. In combination with a cognate peptide epitope the application of imiquimod mediates a strong primary immune response [8], [9] leading to tumor protection in a prophylactic model. In therapeutic tumor settings TCI merely mediates a delay in tumor growth in transplanted EG.7 tumors [10] as well as in B16 melanoma models [11], [12]. An important step in the development and improvement of TCI is an enhanced understanding of the underlying mechanisms and limitations of TCI with imiquimod. This might elicit the opportunity to increase inflammatory factors or circumvent suppressing influences. Concerning the activation of SALT after TCI with imiquimod we and others have demonstrated that increased numbers of CD11c^+^ dermal DCs migrating out of the skin into the draining lymph nodes where we could also detect an induction of IL-12 [13]. In terms of limiting factors regulatory T cells (T_reg_) may hold a central position as their suppressive function has been shown to be limiting in vaccinations against tumor antigens [14], [15]. One well-described route of suppression mediated by T_reg_ is via the release of IL-10, which influences the production of proinflammatory cytokines by DC and macrophages.

In our present work we explored the role of T_reg_ and IL-10 in our transcutaneous vaccination setting. Here we show a strong influence of T_reg_ and also of the suppressive cytokine IL-10 on the induced immune response. The negative influence of T_reg_ seems to be independent of the decreasing effects of IL-10 as they are not the IL-10 producers in this system. To narrow the source of IL-10 further down we investigated in the role of B cells. A substantial participation of B cells in this vaccination setting, either as IL-10 producers in the case of regulatory B cells or as distributers of inflammatory cytokines after imiquimod application, could be excluded.

## Results

### Regulatory T cells suppress TCI-induced CTL responses

**Figure 1:**
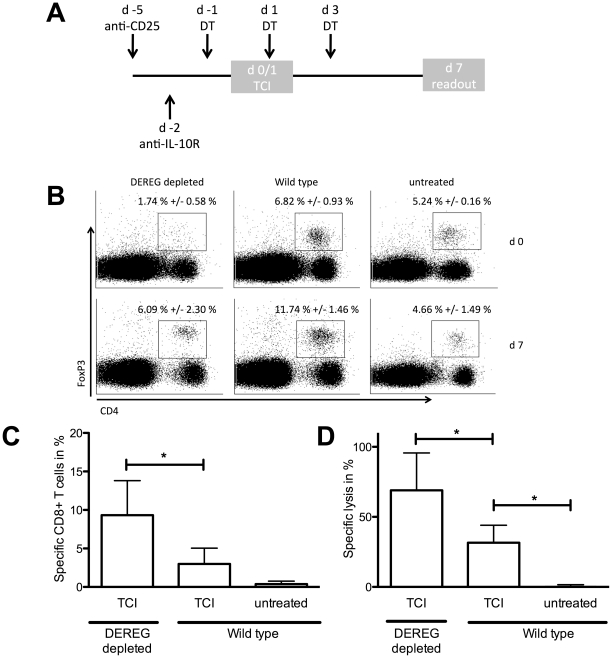
FoxP3^+^ regulatory T cells inhibit TCI-induced immune response. (A) Transcutaneous immunizations with imiquimod were performed at days 0 and 1 with Aldara (50 mg together with 100 µg SIINFEKL peptide). Mice were additionally treated with diphtheria toxin (days -1, 1 and 3), anti-CD25 (day -5) or anti-IL-10R (day -2) as indicated. DEREG mice were depleted of FoxP3^+^ regulatory T cells by intraperitoneal administration of diphtheria toxin (1 µg). Percentages of CD4^+^FoxP3^+^ T_reg_ were assessed at days 0 and 7 (B). Specific CD8^+^ T cells (C) and cytolytic activity (D) were quantified by flow cytometric analysis 7 days after immunization. The depicted results are cumulative from three independent experiments with three mice per group. (*) Significant difference (p<0.05) by Mann-Whitney test.

Regulatory T cells (T_reg_) play a central role in maintaining peripheral tolerance by restricting immune responses. To understand the underlying mechanisms and advance the vaccination strategy we were interested in the influence of T_reg_ in the described immunization protocol. To this end, we immunized mice (DEREG), transgenic for the diphtheria toxin receptor under the FoxP3 promoter. By administration of diphtheria toxin (DT) FoxP3^+^ cells can be specifically ablated. FoxP3-sufficient and - depleted mice were immunized with transcutaneous immunization (Figure 1). To control for the efficacy of DT administration, we performed FoxP3-stainings of blood samples at the day immunization started (d 0) and at the read out time point (d 7). Injection of DT led to a decreased population of CD4^+^FoxP3^+^ T cells (1.74%, +/- 0.58%) compared to immunized (6.82%, +/- 0.93%) or untreated (5.24%, +/- 0.16%) wild type mice at d 0. Staining of CD4^+^FoxP3^+^ T cells at d 7 revealed an increased population in the immunized groups being more prominent in the wild type mice (11.74%, +/- 1.46%) compared to the previously depleted DEREG (Figure 1B). Concerning induced T cell response analysis of peptide specific CD8^+^ T cells showed increased numbers in FoxP3-depleted mice (Figure 1C). Compared to wild type ablation of T_reg_ led to an increased lysis of target cells by cytotoxic T cells (Figure 1D).

**Figure 2:**
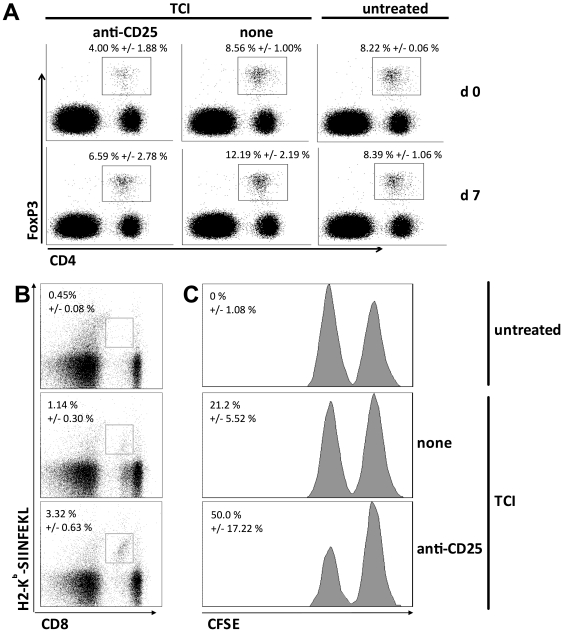
Reduction of regulatory T cell numbers results in enhanced immune responses. C57BL/6 mice were transcutaneously immunized as described before. Were indicated mice received anti-CD25 (clone PC61, 500 µg i.p., day -5) for reduction of regulatory T cell numbers. Percentages of CD4^+^FoxP3^+^ T_reg_ were assessed at days 0 and 7 (A). Specific CD8^+^ T cells (B) were quantified by H2-K^b^-Tetramer staining (percentage of CD8^+^H2-K^b^-SIINFEKL^+^ cells). (C) Cytolytic activity was determined 8 hours after adoptive transfer of peptide-loaded splenocytes. Stated percentage indicates specific lysis of target cells. A cumulative analysis of two independent experiments with three mice per group is shown.

To assess the impact of T_reg_ depletion on TCI in a non-transgenic model and further underline the suppressive capacity of CD25^+^ regulatory T cells, we immunized C57BL/6 wild type mice after depletion of CD25^+^ cells by injection of a specific mAb (clone PC61). Staining of CD4^+^FoxP3^+^ T_reg_ showed a decreased cell population after treatment with anti-CD25 (4.00%, +/- 1.88%) compared to untreated groups (8.56%, +/- 1.00% and 8.22%, +/- 0.06%) at d0 (Figure 2A). At the time of read out, anti-CD25-treated mice showed a slightly decreased amount of CD4^+^FoxP3^+^ T_reg_ (6.59%, +/- 2.78%) compared the untreated control (8.39%, +/- 1.06%). The immunized wild type group again showed an increased population of T_reg_ (12.19%, +/- 2.19%). We determined induction of CTL response by the percentage of peptide-specific T cells as well as the IFNγ-production of blood samples and an *in vivo* cytotoxicity assay to address the lytic capacity of cytotoxic T cells. In line with the previous results T_reg_ depletion increased the number of specific T cells (3.32%, +/- 0.63%) compared to TCI in the non-depleted wild type (1.14%, +/- 0.30%) (Figure 2B). In addition an enhanced *in vivo* cytotoxicity (Figure 2C), shown in a decreased population of peptide-loaded target cells, could be defined (anti-CD25/TCI 50%, +/- 17.22%; TCI 21.2%, +/- 5.52%). These results demonstrate a fundamental role of T_reg_ suppressing or restricting induced CTL responses after immunization at the time of CTL priming rather than in the effector phase.

### IL-10 suppresses immune responses after TCI

**Figure 3:**
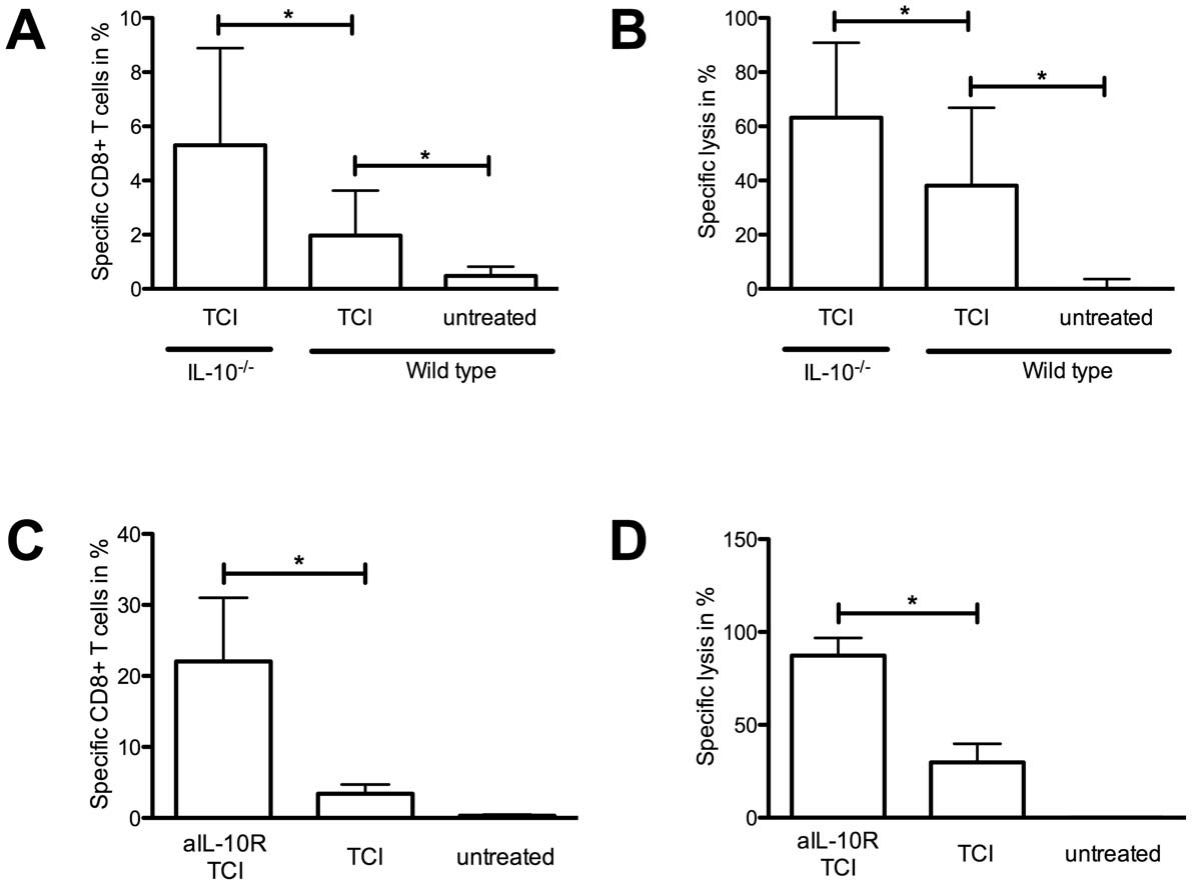
Prevention of IL-10 signalling enhances the induced immune response. IL-10^-/-^ and wild type mice were immunized as described before. (A) Peptide specific T cells as well as (B) lysis of peptide loaded target cells were assessed. C57BL/6 mice were immunized with TCI. Where indicated mice received an intraperitoneal injection of a blocking anti-IL-10-receptor antibody (aIL-10R, 250 µg, d-2). Shown are the results for (C) the quantity of peptide specific T cells, and (D) *in vivo* lysis of transferred target cells. A cumulative analysis of three independent experiments with three mice per group is shown. (*) Significant difference (p<0.05) by Mann-Whitney test.

After demonstrating that T_reg_ suppress TCI-induced CTL responses, we were interested in underlying mechanisms, in particular the contribution of IL-10 since *in vivo* studies show that naturally occurring as well as induced T_reg_ produce IL-10 to control immune responses [16]. To address the role of IL-10 in the context of TCI we first immunized IL-10 deficient mice (Figure 3). 7 days after treatments we analyzed the frequency of peptide specific CD8^+^ T cells and the lytic activity. In the absence of IL-10 we found a high frequency of specific T cells after TCI. Consistent with this, the *in vivo* cytotoxicity assay showed enhanced lytic efficiency compared to wild type controls. To further support the participation of IL-10 in suppression of TCI-induced immune responses using a non-transgenic model, we treated wild type mice as described before with or without prior application of a blocking IL-10-receptor Ab. The results found were in line with those received from the IL-10^-/-^ mice. Bypassing the IL-10 signalling pathway led to increased numbers of peptide specific T cells (Figure 3C) as well as to nearly complete lysis of transferred and peptide loaded target cells (Figure 3D) and enhanced production of IFNγ (data not shown). These results show a substantial participation of the suppressive cytokine IL-10 in diminishing induced CTL responses. Defining the cellular source of IL-10 would contribute to the further understanding and advancing of vaccination protocols.

### Suppression of TCI induced CTL-responses by Regulatory T cells is not mediated by IL-10

**Figure 4:**
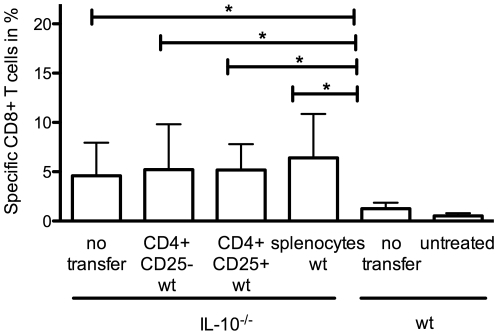
The suppressive capacity of regulatory T cells is not mediated via IL-10. C57BL/6 and IL-10^-/-^-mice were immunized with TCI as described before. Where indicated mice received purified CD4^+^CD25^+^ or CD4^+^CD25^-^ T cells or splenocytes (containing CD4^+^CD25^+^ and CD4^+^CD25^-^)(1×10^6^, d-2) from wild type donors by intravenous injection. After 7 days the amount of H2-K^b^-SIINFEKL^+^CD8^+^ T cells from blood samples was assessed. A cumulative analysis of two independent experiments with three mice per group is shown. (*) Significant difference (p<0.05) by Mann-Whitney test.

The suppressive effects of regulatory T cells may be mediated via the release of IL-10, which also contributes to the regulation of induced CTL-response. To evaluate whether T_reg_ themselves function as the major IL-10 producers, we used IL-10^-/-^ mice and adoptively transferred purified CD4^+^CD25^+^ or only CD4^+^CD25^-^ T cells from wild type mice before immunization. This leads to a situation where only the transferred naturally occurring T_reg_ or CD4^+^CD25^-^ induced T_reg_ are able to produce and release IL-10. To address whether a cooperation between T_reg_ and conventional CD25^-^ T cells is necessary to release suppressive capacities we transferred splenocytes from wild type mice containing with all T cell populations (i.e. CD4^+^CD25^+^ as well as CD4^+^CD25^-^ T cell populations) as control. As shown in Figure 4, neither transfer of purified T_reg_ nor transfer of CD4^+^CD25^-^ T cells nor splenocytes as a whole were able to suppress the enhanced CTL response in IL-10^-/-^ mice to wild type level. This allows the conclusion that in this vaccination model T_reg_ mediate their suppressive capacity mainly via other regulatory functions than IL-10. Alternative cell populations acting as relevant IL-10 producers in this setting need to be determined.

### B cells are not responsible for the release of suppressive IL-10

**Figure 5:**
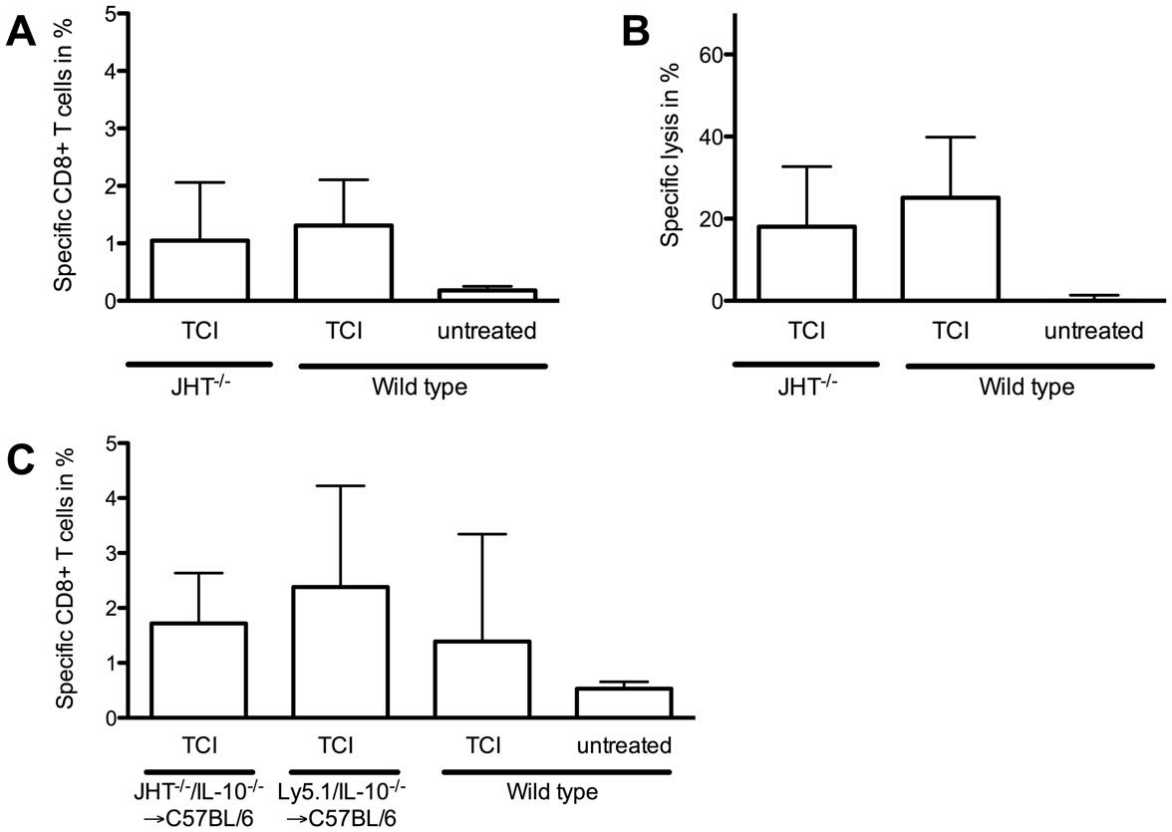
B cells do not have an impact on TCI induced immune response. B cell deficient JHT^-/-^ and C57BL/6 wild type mice were immunized with TCI. Specific CD8^+^ T cells (A) and cytolytic activity (B) were quantified by flow cytometric analysis 7 days after immunization. To assess the role of B cell-derived IL-10 C57BL/6 recipients received bone marrow transplantation of JHT^-/-^ (80 %) and IL-10^-/-^-mice (20 %). Control C57BL/6 recipients were reconstituted with Ly5.1 (80 %) and IL-10^-/-^ (20 %) bone marrow. After complete reconstitution mice were immunized as indicated and the amount of specific CD8^+^ T cells were determined (C). A cumulative analysis of two independent experiments with three mice per group is shown.

An alternative source of IL-10 may be a subpopulation of B cells, called B10 [16]. Those cells are found in the spleens of naïve mice and Yanaba and co-workers could show that they have the capacity to influence T cell activation and inflammatory responses via the release of IL-10 [17]. Also in cases of EAE [18], B cell-produced IL-10 plays an important role in controlling severity of EAE. To evaluate whether B cells are the main IL-10 source in our immunization model we immunized JHT^-/-^ mice that are deficient in B cells [19]. The lack of B cells leads to similar results compared to wild type controls, as demonstrated by the percentage of peptide specific T cells (Figure 5A) and by cytolytic activity (Figure 5B). To further investigate in the role of IL-10 production by B cells we immunized mixed bone marrow chimeras that lack IL-10 production exclusively in B cells (JHT^-/-^/IL-10^-/-^ →C57BL/6). Control chimeras received bone marrow transplantation from wild type and IL-10 deficient donors resulting in a complete repertoire of cells able to produce IL-10 (Ly5.1/IL-10^-/-^ →C57BL/6). The analysis of specific CD8^+^ T cells reveals no participation of B cell-derived IL-10 in this immunization strategy shown in equal amounts of specific CD8^+^ T cells in JHT^-/-^/IL-10^-/-^ →C57BL/6 chimera as well as in the control chimera or wild type group (Figure 5C).

This indicates that also B cells can be excluded as major players of CTL responses induced by our TCI approach, either as enhancers or as counter regulators by the release of IL-10.

## Discussion

To combat infectious or tumor diseases there is a great interest in the development of new and effective vaccination strategies. One promising candidate may be the transcutaneous immunization with imiquimod. For this easy-to-use treatment various information concerning the immune stimulatory capacity could already be accumulated. These include amongst others the release of cytokines and the induction of a potent primary immune response when combined with a CTL-epitope [9], leading to tumor protection in prophylactic models [10].

Targeting the skin as an immunological organ makes it difficult to understand ongoing processes and to annihilate any emerging problems because of the complex interactions of cells in the skin. In terms of the transcutaneous immunization with imiquimod the underlying cellular connections as well as the influencing cytokine-profile is not known in detail. Beside the stimulatory capacity of imiquimod on various cells especially in the skin also regulatory mechanisms are induced. Regulatory CD4^+^CD25^+^ T cells can elicit their suppressive capacity via the release of IL-10 [20], [21] what can be blocked by neutralizing anti-IL-10 Abs [21]. In our present work we wanted to investigate the suppressive mechanisms impeding TCI induced immunity and investigate the role of regulatory T cells and IL-10. We demonstrate, that indeed T_reg_ as well as IL-10 have suppressive functions in this context. Since the release of IL-10 by T_reg_ is an important mechanism of suppression in some models [22], [23] we investigated the role of T_reg_ derived IL-10. As a result we can exclude that a relevant amount of IL-10 after TCI is produced by naturally occurring T_reg_ or induced CD4^+^ CD25^–^ T_reg_ since the transfer of IL-10 sufficient cells in the knock out animals does not restore the wild type situation (Fig. 4). Therefore concerning the mechanisms of T_reg_ mediated suppression of TCI induced CTL activation, other modes of action are obviously important: for example the transfer of cyclic adenosine monophosphate (cAMP) via direct contact of T_reg_ and DC [24], [25], GITR [26] or CTLA-4 [27] need to be investigated in the context of TCI. In addition, TGF-β was found by several groups to be involved in T_reg_-mediated suppression. However, depending on the experimental approach (*in vitro* vs. *in vivo*) and the particular animal model used the importance of TGF-β in T_reg_ mediated suppression is still being controversially discussed [28]. Nevertheless, it was recently shown that T_reg_-derived TGF-β1 suppressed pathogenic T effector cells in a spontaneously occurring T cell-mediated murine model for psoriasis [29]. Given the immense importance of TGF-β in many biological and immunological processes beyond T_reg_ mediated suppression [30], [31], it will remain difficult to assess the true role of TGF-β in T_reg_ without the availability of cell specific knock out animals.

Concerning the alternative sources of IL-10 a number of cell types have to be taken in consideration, for example regulatory B cells (B10) [18]. For this cell population, an important role in EAE has been shown, but little is known about their general embedment in other immune reactions. However a central contribution of B10 cells in terms of regulating TCI-induced immune response can be excluded.

As the suppressive capacity of IL-10 in the context of TCI is axiomatic, further studies are required to identify the relevant sources of IL-10. There are numerous cell types in the skin that are well known IL-10 producers in principle including macrophages [32], keratinocytes [33], mast cells [34] or the recently described CD8^+^ DC [32]. However, the individual role of these cells after imiquimod treatment as relevant IL-10 producers is currently unclear and needs further investigations.

In summary our data provide evidence that induced CTL responses after TCI are subjected not only to conveying but also to suppressing circumstances leading to a complex interplay of various cell types and cytokines. Understanding the underlying mechanisms in more detail would lead to further improvements of vaccination protocols to combat tumors and persistent virus infections.

## Materials and Methods

### Ethics Statement

All animal work performed in this study was conducted according to the national guidelines and was reviewed and confirmed by an institutional review board / ethics committee headed by the local animal welfare officer (Prof. Kempski) of the University Medical Center (Mainz, Germany). The responsible national authority finally approved the animal experiments, which is the National Investigation Office Rheinland-Pfalz (Koblenz, Germany). The Appoval ID assigned by this authority: AZ 23 177-07/G08-1-023).

### Mice

C57BL/6 mice at 6–8 weeks were obtained from the local animal facility of the University of Mainz. DEREG mice (kindly provided by T. Sparwasser) [35], IL-10^-/-^ mice [36] and JHT^-/-^ mice (kindly provided by Ari Waisman) [19] are all on the C57BL/6 background.

### Transcutaneous immunization

Transcutaneous immunizations with imiquimod were described previously [9]. Briefly, after complete removal of dorsal hair with electric clippers 50 mg cream containing 5% imiquimod (Aldara) together with OVA_257–264_ (100 µg in DMSO, kindly provided by Dr Stevanovic, Department Immunology, Institute for Cell Biology, University of Tübingen, Germany) were applied on the skin of anesthetized mice on days 0 and 1. Where indicated anti-IL-10-receptor antibody (250 µg day -2, i.p.) was additionally applied. For regulatory T cell depletion DEREG mice received i.p. injections of diphtheria toxin (1 µg) at days -1, 1 and 3 or C57BL/6 mice were treated with. For adoptive transfer of regulatory T cells (CD4^+^CD25^+^) or CD4^+^ T cells (CD4^+^CD25^-^) cells were purified from C57BL/6 mice as previously described [37]. For generation of bone marrow chimeras C57BL/6 recipients were lethally irradiated (9.5 Gy from Cs^137^ source, OB58-BA, Buchler, Braunschweig, Germany) and reconstituted by transfer of 5×10^5^ bone marrow cells from the indicated donor strains. The animals were maintained under specific pathogen free conditions for 8 weeks before use.

### Flow cytometric analyses and *in vivo* cytotoxicity assay

For flow cytometric analysis the following mAbs (all from eBioscience, Frankfurt, Germany) have been used: APC-Cy7-conjugated anti-CD8 (clone 53-6.7), APC-conjugated CD62L (clone MEL-14), PE-conjugated anti-CD8 (53-6.7). Blood samples were collected after tail vein incision. After a hypotonic lysis step samples were incubated with mAbs on ice. For detection of SIINFEKL-specific T cells samples were stained with H2-K^b^ tetramer. Percentage of specific H2-K^b^-SIINFEKL^+^ cells were determined among gated CD8^+^ T cells. H2-K^b^-SIINFEKL^+^ CD4^-^ CD8^-^ cells were regarded as unspecific stainings. *In vivo* cytolytic activity was assessed by transfer of 2×10^7^ target cells labelled with two different carboxyfluorescein diacetate succinimidyl ester-concentrations (CFSE^high^ and CFSE^low^). The CFSE^low^ population has been additionally loaded with SIINFEKL-peptide. Splenocytes of immunized and control mice were analysed by flow cytometry. All analyses were performed with a LSRII Flow Cytometer and FACSDiva software (BD Pharmingen, Hamburg, Germany).

### Statistical analysis

Different groups were analyzed by Mann-Whitney test for comparison between two groups as indicated. P<0.05 was considered statistically significant.
